# Caregivers serving as confidants: relationship correlates and well-being by care-recipients’ dementia status

**DOI:** 10.1093/geronb/gbaf273

**Published:** 2026-01-02

**Authors:** Meng Huo, Fei Wang, Yongxin Shang, Sarah E Patterson

**Affiliations:** Department of Human Ecology, University of California, Davis, Davis, California, United States; College of Social Work, The University of Tennessee, Knoxville, Knoxville, Tennessee, United States; Brooks School of Public Policy, Cornell University, Ithaca, New York, United States; Institute for Social Research, University of Michigan, Ann Arbor, Michigan, United States

**Keywords:** NHATS, NSOC, Multiplex helper, Family caregiving

## Abstract

**Objectives:**

Family and unpaid caregivers are mainstays of help with daily activities when older adults experience health problems; caregivers can also be relied on as confidants for discussing important matters. It remains unclear what characteristics of caregiving relationships may underlie caregivers’ confidant status, how serving this additional role affects their psychological well-being, and whether these associations vary by the presence of dementia in older adults.

**Methods:**

We used data from the 2017 National Health and Aging Trends Study and the National Study of Caregiving. Participants included 1,694 family or other unpaid caregivers of 1,126 older adults receiving self-care, mobility, or household help due to health and functioning. We estimated logistic regression models for being nominated as a confidant and linear regression models for caregivers’ psychological well-being.

**Results:**

Caregivers who were spouses or children and who reported more positive relationships with the older adults they provided care for were more likely to be nominated as confidants; yet the association involving relationship quality was moderated by older adults’ dementia status. Being a confidant was associated with better well-being among caregivers of older adults without dementia, but not for caregivers of older adults with dementia.

**Discussion:**

Caregivers of older adults with dementia tend to also be a confidant, but serving these multiple roles is only associated with positive psychological well-being for those caring for older adults without dementia. We call for more research to better understand the unique stressors that caregivers experience when serving as confidants for older adults with dementia.

In the United States, more than two out of every five older adults age 65 and older report having at least one type of disability that limits their daily functioning (CDC, 2025). Further, the number of older adults with dementia, a collection of high-need thinking and memory symptoms that can lead to or at least co-occur with disabilities, is expected to grow (Alzheimer’s Association, 2024). With an increase in limitations comes a greater need for receiving help with daily activities, especially those related to health and functioning (Freedman & Spillman, 2014). A majority of older adults, both with and without dementia, receive unpaid care from family members or other acquaintances (e.g., friends or neighbors; Wolff et al., 2025). Unpaid caregivers are typically mainstays of practical assistance with activities such as self-care, mobility, household chores, management of finances, and transportation (National Alliance for Caregiving & AARP, 2020).

Yet, some caregivers may also serve as confidants, known as multiplex helpers (Shang & Patterson, 2024), providing older adults with a trusted space to share personal feelings and discuss private or sensitive matters (Bookwala, 2017; Litwin & Stoeckel, 2014). Most older adults have at least one multiplex helper, often a nuclear family member (i.e., spouse or child), and having a multiplex helper is correlated with older adults’ better subjective well-being (Shang & Patterson, 2024). Although caregiver relationship type and relationship quality have been linked to caregivers’ health and well-being (de Rosa et al., 2025; Huo & Kim, 2023; Lai et al., 2022), there is less clarity on how serving this additional confidant role affects the *caregivers*’ psychological well-being. Moreover, less is known about how other characteristics of the caregiver-care recipient relationship (e.g., quality) are associated with a caregiver’s confidant status.

The current study drew on data from a nationally representative sample of unpaid caregivers (e.g., family and friends helping older adults with self-care, mobility, or household help due to health and functioning) participating in the *National Study of Caregiving (NSOC).* NSOC caregivers are linked with older adults in the *National Health and Aging Trends Study* (NHATS), allowing us to also take into account older adults’ dementia status. Indeed, dementia presents a wide range of complex behavioral and psychological symptoms, which can exacerbate caregiving demands and increase caregiver burden (Freedman et al., 2022; Kasper et al., 2015; Sheehan et al., 2021). Having someone to confide in provides older adults with critical companionship and alleviates their feelings of distress during stressful events (Antonucci et al., 2014). Indeed, older adults tend to increase their reliance on confidants upon receiving a stressful diagnosis, such as a cancer diagnosis (Maunsell et al., 2009), but this may be even more so in the context of dementia. Here, we explicitly compared the effects of serving as a confidant among caregivers of older adults with and without dementia to gain valuable insights into caregivers’ distinct experiences and inform tailored interventions targeting caregivers who may need additional resources.

## Conceptual framework

To identify the relationship correlates and well-being consequences of caregivers’ confidant status, we integrated the hierarchical compensatory model (Cantor, 1979; Penning, 1990) and role theories (Goode, 1960; Kulik et al., 2015; Moen et al., 1992). The hierarchical compensatory model suggests that older adults turn to different sources of support in a hierarchical order, depending on the primacy of their ties to support providers (Cantor, 1979; Penning, 1990). The primacy of ties may be determined by the type and quality of relationships, in that certain ties are more difficult to dissolve and rest on more fundamental, enduring bonds than other ties and may be preferred to fulfill multiple roles in the context of caregiving (e.g., a spouse vs a cousin). As individuals occupy both a caregiver role and a confidant role, the expectations and obligations that come with each role may interact to affect their well-being.

Further, we extended the framework to understand the potentially distinct caregiving experiences and outcomes by care recipients’ dementia status. Socioemotional selectivity theory highlights the overall priority of sustaining pleasant social experiences and close social ties in later life when perceived time left is limited (Carstensen, 2006), but the Strength and Vulnerability Integration (SAVI) Model further predicts that chronic stressors, such as dementia, can prolong older adults’ emotional suffering if conflicts cannot be avoided (Charles, 2010). As such, in the presence of dementia, care recipients may show even stronger preferences per the primacy of social ties when deciding whom to confide in. Coupled with the heightened demands of dementia caregiving, taking on additional confidant roles may place greater strain on caregivers and adversely affect their psychological well-being.

## Relationship correlates of confidant status: type and quality

A growing body of work has recognized that older adults rely on multiple caregivers for support in different domains, and the characteristics of caregiver networks have important implications for older adults’ well-being (Andersson & Monin, 2018; Hu et al., 2023; Leggett et al., 2025). Different caregivers can collaborate and share tasks to better support older adults (Spillman et al., 2020), and it is common for older adults who need care to have “multiplex helpers” who are both caregivers and confidants. In a national sample of older adults with at least one caregiver and one confidant, over three-quarters had at least one multiplex helper (Shang & Patterson, 2024). The extant, yet scarce, research available described the composition of older adults’ multiplex helper networks, pointing out the role that relationship type plays. For instance, among those who were married or partnered, nearly 90% named their spouses/partners as multiplex helpers, whereas for those who were unpartnered (especially the widowed), children were the most common multiplex helpers (Shang & Patterson, 2024). Indeed, spouses and children are typically preferred over other family relatives, friends, neighbors, and other acquaintances to fill both roles of caregiver and confidant (Barker, 2002). We sought to replicate the association between relationship type and confidant status at the caregiver level, hypothesizing:

**Hypothesis 1a:** Caregivers who were nuclear family members (spouses and children) were more likely to be identified as confidants compared to caregivers who were other relatives or non-relatives.

Being considered as a confidant not only reflects the relationship type but also reflects established trust within a relationship (Antonucci et al., 2014). However, it is less clear how the characteristics of each caregiver’s relationship with care recipient—especially the quality of this relationship—affects their confidant status. Not all relationships of the same type are alike, such that a spouse with whom the older adult has a more positive relationship may be more likely to be relied on for multiple roles compared to a spouse with whom one has a less positive relationship. Importantly, in contrast to treating relationship quality as one-dimensional, we considered positive and negative relationship quality separately to identify affection and strain (Mattson et al., 2013; Reblin et al., 2020). These characteristics do not necessarily represent two ends of one spectrum and may independently affect older adults’ preferences for sources of support. We hypothesized:

**Hypothesis 1b:** Caregivers who reported more positive and less negative relationship quality were more likely to be identified as confidants compared to caregivers who reported less positive and more negative relationships.

Yet, these hypothesized associations may be stronger in the presence of dementia, which constitutes a key source of chronic stress. Given age-related vulnerabilities in the context of chronic stress, older adults with dementia may select confidants more strategically and exhibit stronger preferences for those they feel closer to (e.g., nuclear family members, ties with more positive relationship quality). In addition, older adults with dementia often receive help with more tasks and more personal tasks (e.g., bathing) than older adults without dementia (Kasper et al., 2015), and the progression of the disease may impair communication (Small et al., 2000). We hypothesized:

**Hypothesis 1c:** The associations between relationship characteristics (type and quality) and confidant status are stronger among caregivers of older adults with dementia vs caregivers of older adults without dementia.

## Caregivers as confidants and psychological well-being

Most work has focused on how older adults benefit from having someone to confide in (Bookwala, 2017; Connidis & Davies, 1990; Litwin & Stoeckel, 2014; Shang & Patterson, 2024), but we know little about how serving as a confidant affects caregivers’ psychological well-being. Caregiving per se can be stressful, but increasing research has identified positive aspects of caregiving. In most qualitative studies, caregivers described gains related to providing companionship, being relied on, and getting to know care recipients better (Lloyd et al., 2016)—all of which reflect caregivers’ responsibilities and roles as confidants. We considered competing hypotheses based on role theories.

On the one hand, role enrichment theory assumes that serving multiple roles can lead to positive outcomes, such as an increased sense of meaning in life (Kulik et al., 2015; Moen et al., 1992). It is possible that acting as confidants helps caregivers better understand older adults’ needs and fulfill their caregiver roles. Besides, psychological research on friendships has found that providing emotional support strengthens the association between providing instrumental support and feelings of happiness (Morelli et al., 2015). This leads to the following hypothesis:

**Hypothesis 2a (role enrichment):** Serving as a confidant is associated with better psychological well-being of caregivers.

By contrast, role strain theory emphasizes the limited amount of time and energy each individual has, suggesting that serving additional roles may create extra stress and reduce caregivers’ psychological well-being (Goode, 1960). For instance, dual responsibilities, such as work and caregiving, can lead to role overload and thus compromise caregivers’ well-being (Patterson et al., 2023). Despite the limited evidence on the role strain effects of serving an additional confidant role among older adults, a study on caregiver expectations suggests that adult children who were expected by their mothers to provide multiplex support (e.g., being the emotionally close child and also being a confidant) in the future experienced more depressive symptoms (Suitor et al., 2018). As such, there can be a competing hypothesis:

**Hypothesis 2b (role strain):** Serving as a confidant is associated with worse psychological well-being of caregivers.

Caregivers of older adults with dementia typically devote more hours to their responsibilities and experience greater demands, challenges, and role overload than caregivers of older adults without dementia (Freedman et al., 2022; Sheehan et al., 2021; Turner et al., 2023). Serving as a confidant requires additional time and energy, which may further exacerbate caregivers’ struggles in the context of dementia. Moreover, reciprocal communication wanes as cognition deteriorates, so do the emotional rewards of serving as confidants and engaging in intimate discussions (Aneshensel et al., 1995). Thus, we hypothesized:

**Hypothesis 2c:** Caregivers of older adults with dementia would experience poorer psychological outcomes due to serving as confidants compared to caregivers of older adults without dementia.

## Other factors

We considered other factors that may affect the association between being a multiplex helper and caregivers’ psychological well-being: sociodemographic characteristics and caregiving-related characteristics (hours of care, being the only caregiver, and duration of caregiving). Age and marital status are highly correlated with the relationship type of helpers, such that spousal caregivers tend to be older than child caregivers, and only partnered older adults can have spousal caregivers. Older, married, healthier, and better-educated adults typically report better psychological well-being (Charles, 2010). Women are more involved in close ties with both family and friends and thus more likely to be identified as confidants (Bookwala, 2017), but women are also more likely than men to report worse health outcomes from caregiving (Pinquart & Sörensen, 2006). People from racial/ethnic minority groups (e.g., African Americans, Asian Americans, Hispanic/Latinx Americans) may prefer keeping smaller networks and including more kin ties (Dong & Chang, 2017; Lin, 2025; Marquez et al., 2014); therefore, it is possible that minority caregivers serve more roles than their non-Hispanic White counterparts. Black caregivers typically provide more hours of caregiving but report more gains and better psychological well-being than White caregivers, whereas Latinx caregivers tend to report lower physical well-being than White caregivers (Fabius et al., 2020; Liu et al., 2021; Pinquart & Sörensen, 2005). Spousal caregivers tend to co-reside with older adults who have disabilities, but coresidence status, regardless of relationship type, may intensify caregiving responsibilities and affect caregivers’ well-being (Zarit et al., 2019). Hours of care likely reflect demands and may affect their psychological well-being. Relatedly, solo caregivers who do not have others to share care tasks may face more caregiving burden and have worse well-being (Ornstein et al., 2019). The duration of caregiving also matters, in that current and long-term caregiving were both associated with greater depression risk (Capistrant et al., 2014). Caregivers of older adults with dementia also tend to report longer duration of caregiving (Wolff et al., 2025).

## Methods

### Data and study sample

We used data from the NHATS and the supplemental NSOC. NHATS began in 2011 with a nationally representative sample of Medicare beneficiaries aged 65 and older and has been fielded annually. Some of these older adults received unpaid help with activities of daily living (e.g., mobility, self-care, household activities, money matters, medical activities, transportation). People who provided such help with at least one activity in the past month, including care recipients’ family members and friends, were identified as caregivers. Older adults nominated as many caregivers as there were, and selective caregivers were contacted to participate in NSOC. NSOC included cross-sectional samples of caregivers in 2011, 2015, 2017, and 2021 through 2024 (Freedman et al., 2019). We used data collected in 2017, which included the largest sample of caregivers (2,652 caregivers of 1,697 older adults receiving care). Further, it allowed us to test our hypotheses independent of the complex impact of the COVID-19 pandemic on family caregiving (Beach et al., 2021; Cohen et al., 2020).

Among the 2,652 caregivers, 328 were excluded because their care recipients passed away and/or they did not help in the last month prior to the interview, 376 were excluded because care recipient data were provided by proxy respondents (thus, no confidant was nominated), and an additional 254 caregivers were excluded due to missing data on at least one key variable. The final analytic sample included 1,694 caregivers of 1,126 older adults who were not missing on any variables of interest.

### Measures

#### Caregiver confidant status

In each wave of NHATS, older adults were asked to identify up to 5 people they talked with most often about important things over the last year (Freedman et al., 2024). The definition of “important things” in the item wording included good or bad things that happened to them, problems they were having, or important concerns they might have. This approach to social network aligns with traditional methods of egocentric social network data collection and is comparable to the National Social Life Health & Aging Project Confidant Roster (Cornwell et al., 2009). The people they nominated were considered as confidants. We matched these people to caregivers in NSOC and generated an indicator of confidant status among caregivers (1 = *yes*, 0 = *no*).

#### Care recipient dementia status

Following Kasper et al. (2013), we relied on three types of information from NHATS to identify older adults with dementia: (a) self or proxy report of whether older adults had ever been told that they had dementia or Alzheimer’s disease, (b) AD8 Dementia Screening Interview, and (c) cognitive test scores. The AD8 was administered to proxy respondents and included eight items assessing memory, temporal orientation, judgment, and function. Older adults completed cognitive tests assessing their memory, orientation, and executive function. We used the latest coding provided in the 2024 technical report to classify older adults participating in NHATS into three groups: probable dementia, possible dementia, and no dementia. Older adults were assigned to the probable dementia group if they self-reported having received a diagnosis or if they received a score no higher than 1.5 standard deviations (*SD*) below the mean in at least two domains of cognitive tests. Older adults were assigned to the possible dementia group if they received a score no higher than 1.5 *SD* below the mean in at least one cognitive domain. We then adopted the broad definition of dementia and combined probable dementia and possible dementia to generate an indicator of dementia status for each care recipient (1 = *living with probable or possible dementia*, 0 = *living with no dementia*).

#### Relationship characteristics between caregivers and care recipients

Older adults listed each caregiver’s relationship to them during the NHATS interview, based on which we assigned caregivers into four types: spouse/partner, children (daughter/son/daughter-in-law/son-in-law/stepdaughter/stepson), other relatives (e.g., siblings, grandchildren, other relatives), and non-relatives (e.g., friends, neighbors).

Caregivers in NSOC rated the quality of relationships they had with older adults (Huo & Kim, 2023). Positive items included: (a) How much do you enjoy being with [the older adult]? and (b) How much does [the older adult] appreciate what you do for [him/her]?. Negative items included: (a) How much does [the older adult] argue with you?, and (b) How often does [the older adult] get on your nerves?. All items were rated on a scale from 1 (*a lot*) to 4 (*not at all*), but we reverse-coded participant responses such that higher mean scores for positive and negative items indicated more positive and more negative relationships, respectively (*α*_positive_ = 0.56, *α*_negative_ = 0.75).

#### Other factors

We also adjusted for caregivers’ sociodemographic characteristics and hours of care. Caregivers reported their age in years, gender as 1 (*male*) or 0 (*female*), self-rated health as 1 (*poor*) to 5 (*excellent*), and marital status recoded as 1 (*married/living with a partner*) or 0 (*separated/divorced/widowed/never married*). As for education, caregivers indicated the highest degree or level of school they completed, on a scale including 1 (*no schooling completed*), 2 (*1st–12th grade*), 3 (*9th–12th grade*), 4 (*high school graduate*), 5 (*vocational, technical, business, or trade school certificate or diploma*), 6 (*some college but no degree*), 7 (*associate’s degree*), 8 (*Bachelor’s degree*), and 9 (*Master’s, professional, or doctoral degree*). We coded race/ethnicity into four categories: non-Hispanic White, non-Hispanic Black, Hispanic, and other non-Hispanic races (e.g., American Indian/Asian/Native Hawaiian/Pacific Islander). We also calculated the number of hours each caregiver contributed in the last month, based on caregivers’ self-reports in the NSOC (Freedman et al., 2014). Network contexts are measured as a set of indicators capturing whether the caregiver serves each role (e.g., caregiver, confidant, multiplex helper) alone or together with other network members. We relied on the NHATS Other Person File to count the total number of caregivers for each care recipient and identify the NSOC observation as the “only caregiver” if the care recipient only had one caregiver. An only confidant was identified when a caregiver was the only confidant that their older adult care recipient nominated. An only multiplex helper was identified when a caregiver was the only caregiver also nominated as a confidant by their care recipient (i.e., the care recipient might nominate other confidants, but those confidants were not caregivers). Finally, we generated a categorical variable to assess the duration of caregiving based on caregivers’ reports on when they began providing care: less than 1 year, 1–4 years, and 5 years or more.

#### Caregiver psychological well-being

Caregivers indicated how often they felt (a) cheerful, (b) calm and peaceful, (c) full of life, (d) bored, (e) lonely, and (f) upset in the last month (Palaza et al., 2024), on a scale from 1 (*everyday*) to 5 (*never*). We reverse-coded the positively worded items and calculated a mean score per participant (*α* = 0.78), such that a higher score indicated better psychological well-being.

### Analytic strategy

We computed descriptive statistics of the full sample, compared caregivers by care recipients’ dementia status using independent samples t tests and chi-squared tests, and ran bivariate correlations among key variables (caregivers’ confidant status, care recipients’ dementia status, relationship characteristics, and psychological well-being). We use SAS 9.4, adjusting for sample weights (multiple caregivers per care recipient) and survey design, to make estimates nationally representative (Freedman et al., 2019). Our first set of hypotheses focused on the association between relationship characteristics (type and quality) and caregivers’ confidant status. Because confidant status was a binary outcome variable (1 = *yes*, 0 = *no*), we estimated logistic regressions using PROC SURVEYLOGISTIC to predict the likelihood of caregivers serving as confidants. The second set of hypotheses examined the association between caregivers’ confidant status and their psychological well-being. We estimated linear regressions using PROC SURVEYREG.

To assess the moderating effect of care recipients’ dementia status, we reran the analyses above, adding interaction terms of each predictor (relationship type, relationship quality, confidant status) × care recipients’ dementia status. We grand mean centered all continuous variables. All significant moderations were further explored using simple slopes analyses, such that we assessed separate associations among caregivers of older adults with dementia and caregivers of older adults without dementia.

## Results

### Descriptive statistics and bivariate correlations

Table 1 presents the weighted descriptive statistics of the total sample of 1,694 caregivers examined in this study and comparison tests between caregivers of older adults with dementia and those for older adults without dementia. Among all, 21% of caregivers were spouses/partners of care recipients, 51% were children, 18% were other relatives, and 10% were non-relatives. Caregivers reported high levels of positivity (3.86 out of 4.00) and low levels of negativity (2.00 out of 4.00) when commenting on the quality of their relationships with older adults receiving care, and experienced medium to high levels of psychological well-being (3.89 out of 5.00). In total, 1,068 (62%) caregivers were considered as confidants by older adults; 257 (13%) were the only confidant, and 509 (29%) were the only multiplex helper for their care recipients.

**Table 1.** Weighted sample characteristics of caregivers.

Characteristics	Total sample of caregivers (*N *= 1,694)			For older adults without dementia (*n *= 1,044)		For older adults with dementia (*n *= 650)		
	*M/%*	*SE*	Range	*M/%*	*SE*	*M/%*	*SE*	*t* or *χ^2^*
**Confidant status, %**	62			63		61		0.54
**Relationship type**								
** Spouse/partner, %**	21			24		15		18.48***
** Child, %**	51			48		57		11.58**
** Other relative, %**	18			17		19		0.67
** Non-relative, %**	10			11		9		0.73
**Relationship quality**								
** Positive relationship quality**	3.86	0.01	1–4	3.88	0.01	3.81	0.02	3.17**
** Negative relationship quality**	2.00	0.03	1–4	2.00	0.04	2.00	0.04	−0.02
**Psychological well-being**	3.89	0.02	1–5	3.89	0.02	3.88	0.04	0.35
**Sociodemographic characteristics**								
**Age**	59.28	0.56	17–96	58.94	0.74	60.07	0.80	−1.02
**Education**	6.00	0.09	1–9	6.04	0.11	5.90	0.11	0.99
**Self-rated health**	3.49	0.04	1–5	3.51	0.05	3.46	0.05	0.75
**Caregiving hours last month**	57.93	2.31	1–720	52.78	2.97	69.89	4.08	−3.18**
**Network contexts^a^**								
** Only caregivers**	14			16		11		6.26
** Only confidant**	13			12		16		4.49
** Only multiplex helper**	29			30		27		1.22
**Caregiving duration**								
** Less than 1 year**	12			12		12		0.01
** 1–4 years**	36			37		33		3.04
** 5 years or more**	52			51		55		3.02
**Male, %**	36			38		32		4.71
**Married, %**	65			65		65		0.00
**Coresident, %**	41			42		38		1.78
**Race/ethnicity^b^**								
** Non-Hispanic White, %**	76			79		71		13.23*
** Non-Hispanic Black, %**	13			11		16		8.72*
** Hispanic/Latinx, %**	8			7		11		9.27
** Other races (non-Hispanic), %**	3			3		2		2.38

*Note.* Data are from the 2017 *NHATS* and *NSOC*. Range was only reported for continuous variables. All the values presented are adjusted by NSOC sample weights.

a Proportions of caregivers who were the only caregiver, confidant, and multiplex helper for their older adult care recipients.

b The percentages in the total sample column do not add up to 100% due to rounding.

* *p* < .05.

** *p* < .01.

*** *p* < .001.

A caregiver was more likely to also serve as a confidant (thus a multiplex helper) if the care recipient had a smaller caregiver network or a larger confidant network. Supplementary Table 1 (see online supplementary material) further revealed bivariate correlations among the key variables for hypothesis testing: caregiver confidant status, care recipient dementia status, caregiver-care recipient relationship correlates, and caregiver psychological well-being.

### Hypothesis testing

**Figure 1.**
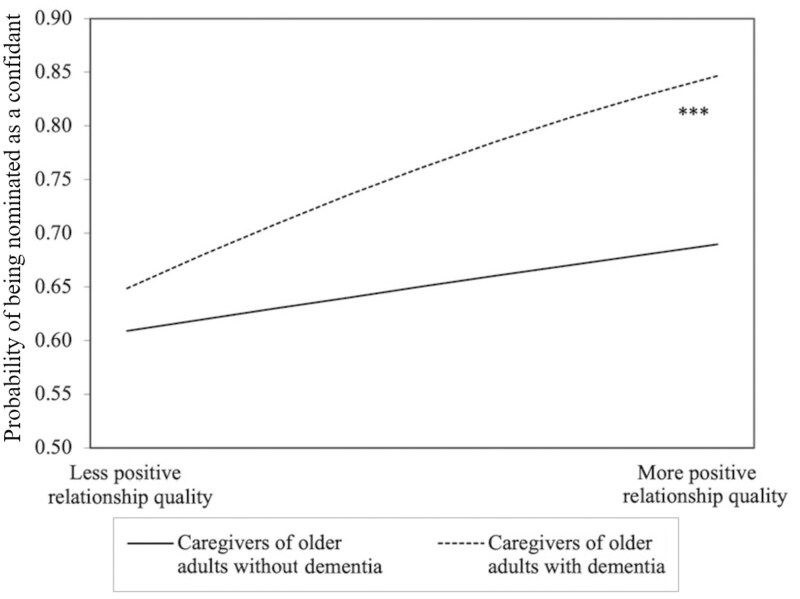
Interaction between positive relationship quality and dementia caregiving status. *Note*. Data are from the 2017 NHATS and NSOC. *N* = 1,694. Simple slopes analysis based on Model 2 in Table 2 (with control variables). Simple slopes are plotted at the means of all continuous variables and the reference level of all categorical variables. More positive and less positive relationship quality reflect 1 *SD* above and below the mean. ***p < .001.

To test Hypotheses 1a–1c, we examined relationship correlates of caregivers’ confidant status using logistic regression models (see Table 2 adjusted models and Supplementary Table 2 for unadjusted models). Caregivers who were spouses/partners or children (compared to other relatives or non-relatives) and who had more positive relationships with older adults receiving care were more likely to be confidants for those older adults (see Model 1 in Table 2). There was no association between negative relationship quality and caregivers’ confidant status. Further pairwise comparisons for relationship type (not shown in the table) revealed no difference between spousal caregivers and child caregivers, as well as between other relative caregivers and non-relative caregivers in the likelihood of being nominated as confidants. When testing the moderation effects (see Model 2 in Table 2), we observed a significant moderation effect of care recipients’ dementia status on the positive association between positive relationship quality and confidant status. Simple slopes analysis suggested that this positive association was only evident among caregivers of older adults with dementia (*p* < .001), but not among caregivers of older adults without dementia (*p* = .084; see Figure 1). That is, among caregivers of an older adult with dementia, having a more positive relationship quality with the older adult was associated with being nominated by the older adult as a confidant, but this association was not significant among caregivers of older adults without dementia.

**Table 2.** Logistic regression testing relationship correlates of caregivers’ confidant status.

Variable	Model 1: Main Effect			Model 2: Moderation Effect		
	*B*	*SE*	OR	*B*	*SE*	OR
**Intercept**	0.66	0.43		0.62	0.45	
**Relationship type**						
** Spouse or partner**	(Ref.)			(Ref.)		
** Child**	−0.15	0.33	0.86	−0.09	0.35	0.91
** Other relative**	−0.94*	0.41	0.39	−0.72	0.44	0.49
** Non-relative**	−1.14**	0.41	0.32	−1.20*	0.47	0.30
**Relationship quality**						
** Positive relationship quality**	0.83***	0.22	2.30	0.48	0.30	1.62
** Negative relationship quality**	0.05	0.11	1.06	−0.05	0.13	0.95
**Dementia caregiving status**	−0.07	0.17	0.93	0.54	0.55	1.72
**× Spouse or partner**	–	–	–	(Ref.)		
**× Child**	–	–	–	−0.56	0.59	0.57
**× Other relative**	–	–	–	−1.11	0.63	0.33
**× Non-relative**	–	–	–	−0.15	0.74	0.86
**× Positive relationship quality**	–	–	–	1.00*	0.45	2.72
**× Negative relationship quality**	–	–	–	0.37	0.23	1.45
**Sociodemographic characteristics**						
** Age**	0.03***	0.01	1.03	0.03***	0.01	1.03
** Male**	−0.71***	0.16	0.49	−0.71***	0.16	0.49
** Education**	0.12**	0.04	1.13	0.13**	0.04	1.13
** Self-rated health**	0.02	0.08	1.02	0.01	0.08	1.01
** Married**	0.22	0.18	1.25	0.22	0.18	1.25
** Coresident**	0.33	0.22	1.40	0.31	0.22	1.37
** Race/ethnicity**						
** Non-Hispanic White**	(Ref.)			(Ref.)		
** Non-Hispanic Black**	−0.56**	0.18	0.57	−0.57**	0.18	0.57
** Hispanic/Latinx**	−0.08	0.31	0.93	−0.07	0.31	0.93
** Other races (Non-Hispanic)**	−0.10	0.46	0.90	−0.09	0.48	0.92
** Caregiving hours**	0.00**	0.00	1.00	0.00**	0.00	1.00
** Only caregiver**	0.77**	0.28	2.17	0.73**	0.27	2.07
** Caregiving duration**						
** Less than 1 year**	(Ref.)			(Ref.)		
** 1–4 years**	0.35	0.25	1.41	0.34	0.26	1.40
** 5 years or more**	0.46	0.24	1.58	0.43	0.24	1.54

*Note*. *N *= 1,694. *B* = unstandardized coefficient. *SE* = standard error. OR = odds ratios. Data are from the 2017 *NHATS* and *NSOC.* Both models are adjusted by NSOC sample weights.

* *p* < .05.

** *p* < .01.

*** *p* < .001.

**Figure 2.**
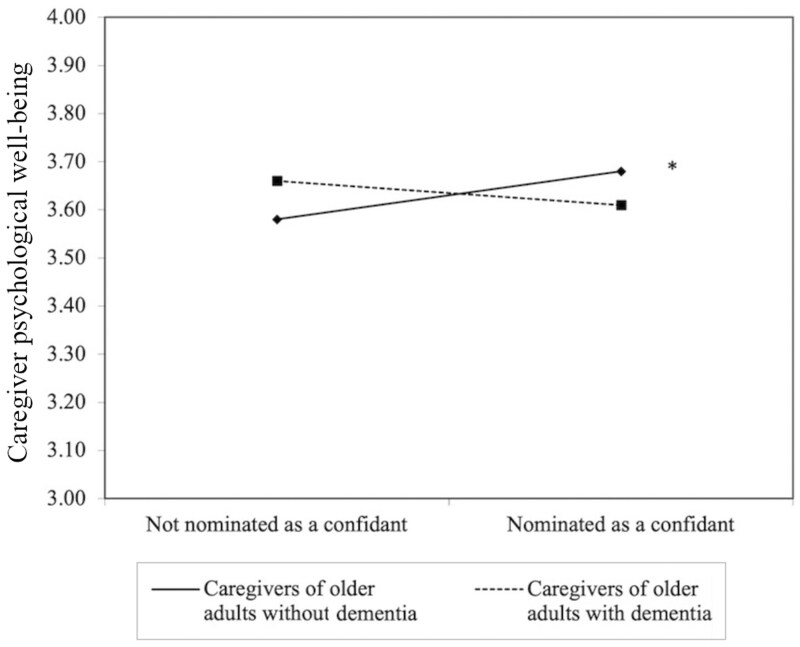
Interaction between caregiver confidant status and care recipient dementia status. *Note*. Data are from the 2017 NHATS and NSOC. *N* = 1,694. Simple slopes analysis based on Model 2 in Table 3 (with control variables). Simple slopes are plotted at the means of all continuous variables and the reference level of all categorical variables. *p < .01.

To test Hypotheses 2a–2c, we examined how caregivers’ confidant status was associated with their psychological well-being (see Table 3 for adjusted models and Supplementary Table 3 for unadjusted models). There was no significant main effect of being a confidant on caregivers’ psychological well-being, but we observed a significant moderation by care recipients’ dementia status. Simple slopes analysis revealed that being a confidant was associated with better psychological well-being among caregivers of older adults without dementia (*B *= 0.10, *p* = .034), whereas the association was not significant among caregivers of older adults with dementia (*B *= −0.05, *p* = .432; see Figure 2).

**Table 3.** Linear regressions examining the association between caregivers’ confidant status and psychological well-being.

Variable	Model 1: Main effect		Model 2: Moderation effect	
	*B*	*SE*	*B*	*SE*
**Intercept**	3.23***	0.26	3.58***	0.11
**Confidant status**	0.05	0.04	0.10*	0.05
**Dementia caregiving status**	−0.01	0.04	0.08	0.06
**× Confidant status**	–	–	−0.15*	0.07
**Relationship characteristics**				
** Spouse or partner**	(Ref.)		(Ref.)	
** Child**	0.18*	0.07	0.19**	0.07
** Other relative**	0.17	0.10	0.18	0.10
** Non-relative**	0.05	0.09	0.06	0.09
** Positive relationship quality**	0.19**	0.06	0.20***	0.06
** Negative relationship quality**	−0.17***	0.02	−0.17***	0.02
**Sociodemographic covariates**				
** Age**	0.01***	0.00	0.01***	0.00
** Male**	0.07	0.04	0.07	0.04
** Education**	0.01	0.01	0.01	0.01
** Self-rated health**	0.18***	0.02	0.18***	0.02
** Married/partnered**	0.08	0.04	0.08	0.04
** Coresident**	0.06	0.05	0.06	0.05
** Race/ethnicity**				
** Non-Hispanic White**	(Ref.)		(Ref.)	
** Non-Hispanic Black**	0.10*	0.04	0.10*	0.04
** Hispanic/Latinx**	0.11	0.08	0.11	0.08
** Other races**	−0.13	0.17	−0.13	0.17
** Caregiving hours**	−0.00	0.00	−0.00	0.00
** Only caregiver**	0.12*	0.05	0.12*	0.05
** Caregiving duration**				
** Less than 1 year**	(Ref.)		(Ref.)	
** 1–4 years**	−0.04	0.06	−0.04	0.06
** 5 years or more**	0.01	0.06	0.02	0.06

*Note*. *N *= 1,649. *B* = Unstandardized Coefficient. *SE* = Standard Error. Data are from the 2017 *NHATS* and *NSOC.* Both models are adjusted by NSOC sample weights.

* *p* < .05.

** *p* < .01.™

*** *p* < .001.

### Sensitivity analyses

We ran a series of sensitivity tests below and reported significant results. Non-significant results are available upon request. We re-estimated the H2a-H2c analyses treating as predictors (a) whether the caregiver was the only multiplex helper for their care recipient (yes/no) and (b) whether the caregiver was one of the multiple multiplex helpers for their care recipient (yes/no). When an older adult has multiple multiplex helpers, they have multiple caregivers also serving as confidants. Care recipients’ dementia status moderated the association between caregiver being the only multiplex helper and psychological well-being, such that this association was only evident when care recipients did not have dementia (*B *= 0.13, *p* = .027). This moderating effect was consistent with the one reported in the main results.

We also examined items of the psychological well-being measure as separate outcomes and found significant interaction effects on the extent to which caregivers felt cheerful and bored. Specifically, being a confidant was associated with feeling more cheerful (*B *= 0.19, *p* = .008) and less bored (*B *= −0.23, *p* = .007) only when caregivers had recipients without dementia. Being the only multiplex helper was associated with feeling more cheerful (*B *= 0.25, *p* = .002) and less bored (*B *= −0.28, *p* = .005) among caregivers of older adults without dementia. Yet, for caregivers whose recipients had dementia, being the only multiplex helper was associated with feeling less cheerful (*B *= −0.23, *p* = .023).

Out of the 1,694 caregivers in 2017, 536 were identified as caregivers in 2015 as well, forming a longitudinal sample for our sensitivity analysis. In this sample, 63 (12%) were confidants only in 2015, and 70 (13%) were confidants only in 2017. In terms of their care recipients’ dementia status, 35 (7%) caregivers had a care recipient with dementia only in 2015, and 93 (17%) cared for an older adult with dementia only in 2017. Logistic regressions revealed that a spousal/partner caregiver was most likely to be a confidant in both 2015 and 2017 (*n *= 304), followed by a child caregiver and then an other-relative caregiver. We re-estimated the Hypotheses 2a–2c analyses, focusing on the 536 caregivers and adjusting for their psychological well-being in 2015. Findings (main and moderation effects) were the same as those reported above, even with a reduced sample size. Baseline confidant status was not associated with psychological well-being at follow-up.

Building on the main results reported above, we estimated three-way interaction tests and found a significant interaction of caregiver confidant status × caregiver-care recipient positive relationship quality × care recipient dementia status (*B *= 0.64, *p* = .002; Supplementary Figure 1). Simple slopes analysis further revealed that being a confidant was positively associated with the psychological well-being of caregivers who had care recipients without dementia and reported low positive relationship quality (*B *= 0.18, *p* = .022), but this association was negative for caregivers who had care recipients with dementia and reported low positive relationship quality (*B *= −0.18, *p* = .012). The associations were not significant when caregivers reported a high positive relationship quality.

## Discussion

Older adults turn to their social networks for a variety of assistance (Litwin & Stoeckel, 2014; McPherson et al., 2006), but their sub-network members who serve different functions may overlap, especially when health needs arise or when they have dementia (Shang & Patterson, 2024; Spillman et al., 2020). Recent work has revealed how older adults may benefit from overlaps between their confidant networks and caregiver networks (Shang & Patterson, 2024), but we made unique contributions to the literature by shifting the attention to caregiver-specific characteristics (e.g., relationship quality), well-being, and variation by whether the older adult has dementia or not. Using a nationally representative sample of caregivers helping older adults aged 65 and older, we found that spousal and child caregivers, as well as those reporting more positive relationships with older adults, were more likely to also be nominated as a confidant by these care recipients. There was no main effect of care recipients’ dementia status on caregivers’ confidant status or well-being. Yet, the presence of dementia seems to strengthen the impact of positive relationship quality as older adults identify their confidants and attenuate the psychological benefits that caregivers can derive from serving as confidants. Findings advance our understanding of the caregiver experiences and reveal nuances in their psychological outcomes when serving multiple functions.

### Relationship correlates of confidant status

Extending prior research on older adults’ confidant networks (Connidis & Davies, 1990; Litwin & Stoeckel, 2014), our results support hypotheses 1a and 1b and identify older adults’ clear reliance on family members for confiding about important matters, particularly spouses and children. Some older adults discuss important matters with friends too, but given the small percentage of confidants who were non-relatives (5%), friends who serve as both caregivers and confidants may only take up a small portion of older adults’ support networks. However, despite the fact that older adults with dementia were less likely to have a spousal/partner caregiver in the sample overall, we did not find a significant interaction between the older adult’s dementia status and the caregiver relationship type in the likelihood of being nominated as a confidant. It is possible that what older adults with dementia value is not certain relationship types, but ties with high positive characteristics.

Indeed, our results partially confirmed hypotheses 1b and 1c. There was an association between positive relationship quality and confidant status, and this association was more salient among caregivers of older adults with dementia. In our measures, confidants were nominated by older adults as those they talked to most often about important things, which inherently reflected trust and closeness. The positive relationship quality variable used here measured whether the caregiver enjoyed being with the older adult and whether they felt appreciated by the older adult. Therefore, it makes intuitive sense that caregivers who were also confidants were potentially aware of the trust and thus reported more positive relationships with care recipients than caregivers who were not confidants. Similarly, older adults may prefer to confide in people they feel more positively connected to in the first place, which over time can further strengthen their relationships. In terms of variation by care recipients’ dementia status, a prior study using the NHATS data found that compared to those without dementia, older adults with dementia more often relied on caregivers who assisted with multiple domains of activities, such as medical, household, and mobility (Spillman et al., 2020). Our result added to this work by suggesting that when it comes to emotional support, older adults with dementia may be more selective and place stronger values on the quality of existing relationships, which extends the SAVI model (Charles, 2010). Notably, relationship quality was reported by caregivers and may also indicate their availability and willingness to serve as confidants. Qualitative data on both parties’ perceptions of their relationships and caregivers’ responsibilities may help us further understand the processes underlying this difference between caregivers of adults with and without dementia.

In support of treating positive and negative relationship quality separately, we found that there was no significant association between negative relationship quality and confidant status. Caregiving easily elicits negative or ambivalent feelings about relationships with care recipients (Caputo, 2021; Huo, Gilligan, et al., 2024) but these feelings may not prevent caregivers from serving additional confidant roles. Likewise, negative relationship quality may not prevent older adults from considering certain caregivers as confidants.

### Caregivers as confidants and psychological well-being

We did not observe an overall association between caregivers’ confidant status and their psychological well-being; rather, we observed a significant moderating effect of older adults’ dementia status. There was a positive association only evident in caregivers of older adults without dementia in support of Hypothesis 2c. This finding extends a prior study that used 24-hr time diaries, which tracked caregivers over the course of one day and revealed differential emotional outcomes of socializing with care recipients. Specifically, socializing with care recipients was associated with less stress in caregivers of older adults without dementia but more negative emotional health in caregivers of older adults with dementia (Freedman et al., 2022). In line with the role enrichment theory (Kulik et al., 2015) and the burgeoning literature on positive aspects of caregiving (Lloyd et al., 2016), being the one older adults feel comfortable confiding in may bring caregivers emotional rewards and strengthen their relationship closeness. Indeed, being a confidant seems to be particularly beneficial among caregivers who reported relatively lower positive relationship quality with older adults not living with dementia. The deep, personal discussions may provide those caregivers with unique opportunities to better understand older adults’ concerns, needs, and expectations, which in turn fulfill caregivers’ goals to contribute to older adults’ welfare.

Yet, among caregivers caring for older adults with dementia, serving additional confidant roles may not be purely enjoyable, especially if the caregiver was the only multiplex helper. In fact, sensitivity tests revealed that being the only multiplex helper or a multiplex helper when positive relationship quality was low compromised caregivers’ well-being. Despite the emotional benefits mentioned above, these caregivers tend to face greater demands and provide more intensive care than those caring for older adults without dementia (Kasper et al., 2015; Sheehan et al., 2021; Spillman et al., 2020). Caring for an older adult with dementia involves routine coping with challenges that are uniquely stressful (e.g., older adults’ behavior) and likely create role strain in caregivers (Cerejeira et al., 2012). These challenges, based on role strain theory (Goode, 1960), may drain caregivers’ limited time and energy and create extra stress that can offset the benefits of being confidants. Future research should consider collecting qualitative data to gain more insight into how caregivers handle tasks across multiple domains (both tangible and intangible) in the presence of dementia.

### Limitations and conclusion

Some limitations in this study warrant consideration. Confidants were identified by care recipients in NHATS based on their experiences in the past year. It is unclear whether caregivers were aware of their confidant status and whether they were continuously nominated as confidants. Importantly, caregivers may also confide in their care recipients and enjoy emotional rewards. Caregivers and care recipients can support each other even in the presence of mild-to-moderate dementia (Ali et al., 2023; Huo, Kim, et al., 2024), which may facilitate mutual understanding and mitigate caregiver isolation in the face of caregiving demands. Future research may further explore how caregivers may benefit from confiding in their care recipients. It is also worth noting that we excluded caregivers of older adults who provided proxy responses due to the lack of confidant nomination questions. A large portion of these older adults with a proxy respondent likely were living with dementia, so our study may have provided a biased portrait of older adults’ confidant networks in the presence of dementia. In particular, older adults with severe dementia were not included in our study, and their significant cognitive declines may somewhat limit the level of conversations they can have with caregivers. Future research may include participants with a wider range of dementia severity and consider the stage of cognitive decline as a moderator. The longitudinal sample of caregivers available in NSOC was only fielded in 2015 and 2017, which would reduce the sample size available to investigate transitions in and out of confidant status in relation to caregiver well-being. Future waves of NSOC that are longitudinal could be used to test this. Although our study is correlational in nature and findings should be interpreted with caution, we present findings that show the nuances of relationship quality, confidant status, and caregiver well-being depending on whether their care recipient has dementia. Finally, we focused on the psychological well-being of caregivers in this study. Future research may consider physical health outcomes or use biomarker data related to inflammation and immunity to better examine whether and how the benefits and costs of caregivers’ multiple responsibilities “get under the skin” (Roth et al., 2015; 2019). Such approaches can add nuances to our understanding of caregiving experiences and outcomes.

Despite these limitations, the current study presents a unique investigation focused on caregivers’ characteristics and well-being when serving additional confidant roles. This work extends the burgeoning literature on network multiplexity in the context of caregiving by adding caregivers’ perspectives and advances our understanding of distinct experiences between caring for older adults with dementia and caring for older adults without dementia. Taken with prior research that revealed positive outcomes in older adults having caregivers who were confidants (Shang & Patterson, 2024), this study suggests how caregiving relationships, at least in the absence of dementia, can be mutually beneficial when caregivers serve additional confidant roles. We hope to inform interventions facilitating confidant relationships in the context of caregiving and allocating additional resources to maximize benefits to caregivers of older adults living with dementia.

## Supplementary Material

gbaf273_Supplementary_Data

## Data Availability

The current study drew on data from the NHATS and the NSOC, which are publicly available on the study website (https://www.nhats.org/researcher). The data we used for analysis and our analytic methods are described in detail in the text, and the code will be made available to other researchers upon request. This study did not involve clinical trials and was not preregistered.
