# Supplementary material for: Caregivers serving as confidants: relationship correlates and well-being by care-recipients’ dementia status
Source: J Gerontol B Psychol Sci Soc Sci. 2026 Jan 2;81(3):gbaf273. doi: 10.1093/geronb/gbaf273 (PMC13376961; doi:10.1093/geronb/gbaf273)
Supplement: gbaf273_Supplementary_Data [file gbaf273_supplementary_data.zip › JGSS suppl Huo]

***The Journals of Gerontology, Series B: Psychological Sciences and Social Sciences* Supplementary Material: Huo, Wang, Shang, & Patterson. Caregivers serving as confidants: relationship correlates and well-being by care-recipients’ dementia status.**

Supplementary Table 1. *Bivariate Correlations*

| **Variable** | **1** | **2** | **3** | **4** | **5** | **6** | **7** | **8** | **9** |
| --- | --- | --- | --- | --- | --- | --- | --- | --- | --- |
| 1. Confidant status | — |  |  |  |  |  |  |  |  |
| 1. Care recipient dementia status | -.04*** | — |  |  |  |  |  |  |  |
| 1. Spouse or partner | .23*** | -.09*** | — |  |  |  |  |  |  |
| 1. Child | .03 | .07** | -.57*** | — |  |  |  |  |  |
| 1. Other relative | -.21*** | .01 | -.22*** | -.50*** | — |  |  |  |  |
| 1. Non-relative | -.12*** | -.00 | -.14*** | -.32*** | -.13*** | — |  |  |  |
| 1. Positive relationship quality | .06* | -.07** | .02 | -.05* | .04 | .01 | — |  |  |
| 1. Negative relationship quality | .08** | .03 | .17*** | .04 | -.14*** | -.15*** | -.41*** | — |  |
| 1. Psychological well-being | -.07** | -.06* | -.03 | -.02 | .02 | .04 | .27*** | -.36*** | — |

*Note*: Data are from the 2017 *National Health and Aging Trends Study (NHATS)* and *National Study of Caregiving (NSOC). N* = 1,694. **p* < .05, ***p* < .01, ****p* < .001.

Supplementary Table 2. *Unadjusted Logistic Regression Testing Relationship Correlates of Caregivers’ Confidant Status*

| **Variable** | **Model 1: Main Effect** | | |  | **Model 2: Moderation Effect** | | |
| --- | --- | --- | --- | --- | --- | --- | --- |
|  | ***B*** | ***SE*** | ***OR*** |  | ***B*** | ***SE*** | ***OR*** |
| Intercept | 1.77*** | 0.20 |  |  | 1.71*** | 0.22 |  |
| Relationship type |  |  |  |  |  |  |  |
| Spouse or partner | (Ref.) | | |  | (Ref.) | | |
| Child | -1.20*** | 0.22 | 0.30 |  | -1.14*** | 0.25 | 0.32 |
| Other relative | -2.28*** | 0.27 | 0.10 |  | -2.08*** | 0.33 | 0.12 |
| Non-relative | -1.96*** | 0.32 | 0.14 |  | -2.01*** | 0.38 | 0.13 |
| Relationship quality |  | | |  |  | | |
| Positive relationship quality | 0.68** | 0.21 | 1.97 |  | 0.27 | 0.29 | 1.31 |
| Negative relationship quality | 0.05 | 0.10 | 1.05 |  | -0.08 | 0.12 | 0.92 |
| Dementia caregiving status | 0.07 | 0.16 | 1.07 |  | 0.53 | 0.53 | 1.70 |
| × Spouse or partner | — | — | — |  | (Ref.) | | |
| × Child | — | — | — |  | -0.39 | 0.56 | 0.68 |
| × Other relative | — | — | — |  | -0.89 | 0.60 | 0.41 |
| × Non-relative | — | — | — |  | 0.02 | 0.73 | 1.02 |
| × Positive relationship quality | — | — | — |  | 1.11** | 0.42 | 3.03 |
| × Negative relationship quality | — | — | — |  | 0.44* | 0.21 | 1.55 |

*Note*. Data are from the 2017 *National Health and Aging Trends Study (NHATS)* and *National Study of Caregiving (NSOC). N* = 1,694. B = Unstandardized Coefficient. SE = Standard Error. OR = Odds Ratios. Both models are adjusted by NSOC sample weights.

**p* < .05, ***p* < .01, ****p* < .001.

Supplementary Table 3. *Linear Regressions Examining the Association Between Caregivers’ Confidant Status and Psychological Well-Being*

| **Variable** | **Model 1:**  **Main Effect** | |  | **Model 2:**  **Moderation Effect** | |
| --- | --- | --- | --- | --- | --- |
|  | ***B*** | ***SE*** |  | ***B*** | ***SE*** |
| Intercept | 3.83*** | 0.04 |  | 3.81*** | 0.04 |
| Confidant status | 0.10* | 0.04 |  | 0.14** | 0.05 |
| Dementia caregiving status | -0.01 | 0.04 |  | 0.07 | 0.06 |
| × Confidant status | — | — |  | -0.13 | 0.08 |

*Note*. Data are from the 2017 *National Health and Aging Trends Study (NHATS)* and *National Study of Caregiving (NSOC). N* = 1,649. B = Unstandardized Coefficient. SE = Standard Error. Both models are adjusted by NSOC sample weights.

**p* < .05, ***p* < .01, ****p* < .001.

Supplementary Figure 1. *Interaction Between Caregiver Confidant Status, Caregiver-Care Recipient Positive Relationship Quality, and Care Recipient Dementia Status*


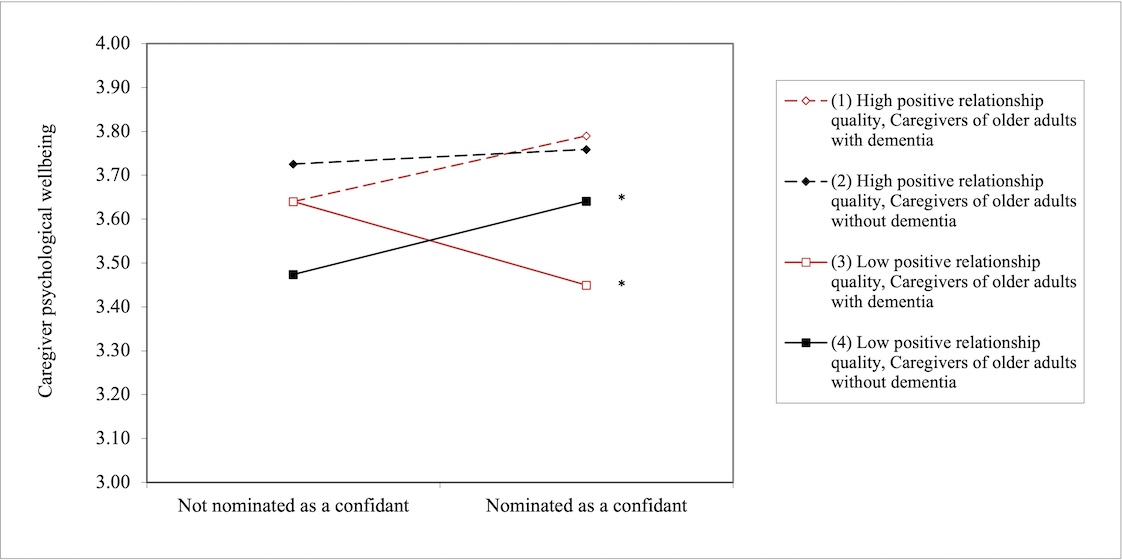


*Note*. Data are from the 2017 National Health and Aging Trends Study (NHATS) and National Study of Caregiving (NSOC). N = 1,694. Simple slopes analysis based on Model 2 in Table 3 (with control variables). Simple slopes are plotted at the means of all continuous variables and the reference level of all categorical variables. More positive and less positive relationship quality reflect 1 standard deviation above and below the mean.

**p* <.05.
